# Influence of Temperature on Growth of Four Different Opportunistic Pathogens in Drinking Water Biofilms

**DOI:** 10.3390/microorganisms11061574

**Published:** 2023-06-14

**Authors:** Paul W. J. J. van der Wielen, Marco Dignum, Agata Donocik, Emmanuelle I. Prest

**Affiliations:** 1 KWR Water Research Institute, 3433 PE Nieuwegein, The Netherlands; 2 Laboratory of Microbiology, Wageningen University & Research, 6708 WE Wageningen, The Netherlands; 3 Waternet, 1090 GJ Amsterdam, The Netherlands; marco.dignum@waternet.nl; 4 Brabant Water, 5223 MA Den Bosch, The Netherlands; agata.donocik@brabantwater.nl; 5 PWN Technologies, 1990 AA Andijk, The Netherlands; emmanuelle.prest@pwnt.com

**Keywords:** opportunistic pathogens, drinking water, biofilm, temperature, climate change

## Abstract

High drinking water temperatures occur due to climate change and could enhance the growth of opportunistic pathogens in drinking water systems. We investigated the influence of drinking water temperatures on the growth of *Pseudomonas aeruginosa*, *Stenotrophomonas maltophilia*, *Mycobacterium kansasii* and *Aspergillus fumigatus* in drinking water biofilms with an autochthonous microflora. Our results reveal that the growth of *P. aeruginosa* and *S. maltophilia* in the biofilm already occurred at 15.0 °C, whereas *M. kansasii* and *A. fumigatus* were able to grow when temperatures were above 20.0 °C and 25.0 °C, respectively. Moreover, the maximum growth yield of *P. aeruginosa*, *M. kansasii* and *A. fumigatus* increased with increasing temperatures up to 30 °C, whereas an effect of temperature on the yield of *S. maltophilia* could not be established. In contrast, the maximum ATP concentration of the biofilm decreased with increasing temperatures. We conclude from these results that high drinking water temperatures caused by, e.g., climate change can result in high numbers of *P. aeruginosa*, *M. kansasii* and *A. fumigatus* in drinking water systems, which poses a possible risk to public health. Consequently, it is recommended for countries with a more moderate climate to use or maintain a drinking water maximum standard temperature of 25 °C.

## 1. Introduction

The growth of microorganisms in drinking water systems is limited in most countries by adding a disinfectant residual during drinking water distribution. In several European countries (e.g., Denmark, the Netherlands, parts of Germany, Belgium, Switzerland, and Austria), however, drinking water is distributed without a disinfectant residual, and microbial growth is controlled by limiting the concentration of biodegradable organic carbon (BDOC) [1]. Nonetheless, microbial growth occurs in drinking water systems, irrespective of whether drinking water is distributed with or without a disinfectant residual. This microbial growth can cause a health threat when opportunistic pathogens, of which *Legionella pneumophila* is the best-known example, multiply in drinking water distribution or premises’ plumbing systems [2].

Surveys of drinking water systems in the Netherlands have detected the opportunistic pathogens *Pseudomonas aeruginosa*, *Stenotrophomonas maltophilia*, *Mycobacterium kansasii* and *Aspergillus fumigatus* in addition to *L. pneumophila* [3,4]. Further work demonstrated that the genotypes of several *P. aeruginosa* and *A. fumigatus* strains and an *S. maltophilia* strain isolated from these drinking water distribution systems were identical to the genotypes isolated from patients, suggesting that the drinking water strains of these two organisms can cause disease in immunocompromised persons [5,6]. Consequently, it is important to investigate possible risk factors that might enhance the growth of these opportunistic pathogens in drinking water systems.

Two trends might impact the occurrence of opportunistic pathogens in drinking water and/or its health risk to humans. First, the number of immunocompromised humans who are vulnerable to infection with opportunistic pathogens is increasing in Western society because of population aging and the longer life span of patients that suffer from serious diseases (e.g., HIV, cancer, cystic fibrosis) [7]. Second, drinking water temperatures are expected to increase in the coming decades due to climate change [8,9], and it is likely that the growth of most opportunistic pathogens in drinking water will be enhanced when water temperature rises for a prolonged time above 20 to 25 °C [10].

The World Health Organization (WHO) recommends a maximum temperature of 25 °C for drinking water at the tap because water at higher temperatures is less palatable and impacts the concentration of several inorganic constituents and chemical contaminants that may affect taste and enhance the growth of microorganisms [11]. A limited number of countries have regulations or guidelines defining drinking water temperature standards [12]. In the Netherlands, the regulatory standard for drinking water temperature is 25 °C, but 0.1% of the samples exceed this 25 °C standard in relatively warm summers [8].

The influence of drinking water temperature on the growth of *L. pneumophila* in the Netherlands has been studied in detail. *L. pneumophila* was capable of growing and competing with indigenous natural microflora or other *Legionella* species at temperatures between 30 and 42 °C [13,14]. In addition, the optimum growth temperature for *P. aeruginosa*, *S. maltophilia*, *M. kansasii* and *A. fumigatus* was observed to be between 25 and 40 °C under optimal laboratory conditions [15,16,17,18,19,20]. It was also noted that *P. aeruginosa* is more prevalent in drinking water in regions with a warm/tropical climate compared to regions with a more temperate climate [21]. The results from these studies suggest that increasing drinking water temperatures might result in increased numbers of opportunistic pathogens in drinking water distribution systems and the cold-water lines of premises’ plumbing systems. However, the influence of the drinking water temperature on the growth of these four opportunistic pathogens under conditions representative of drinking water systems remains unknown. In addition, it is uncertain whether a maximum drinking water temperature standard of 25 °C prevents the multiplication of these opportunistic pathogens in drinking water systems or if the standard can be safely increased.

Maintaining water temperatures below 25 °C at any point in the distribution system and premises’ plumbing systems throughout the year is becoming more challenging for water utilities because of climate change and an increase in district heating systems and electricity networks that can enhance soil and building temperatures. Consequently, acceptance of a drinking water temperature standard higher than 25 °C is preferable, but this should not come at the cost of an increased risk of opportunistic pathogen growth that could threaten public health.

The research objectives of our study were to determine (i) the influence of drinking water temperatures, ranging from 15.0 to 30.0 °C, on biofilm growth of *P. aeruginosa*, *S. maltophilia*, *M. kansasii* and *A. fumigatus* in naturally developed drinking water biofilms and (ii) whether a drinking water temperature standard of 25 °C could be increased without enhancing the risk of higher numbers of these opportunistic pathogens in drinking water systems.

## 2. Materials and Methods

### 2.1. Microbial Strains

The experiments were conducted with strains of *P. aeruginosa* (strain M103998), *S. maltophilia* (strain M143212) and *A. fumigatus* (strain M142650-2) that were isolated from drinking water distribution systems in the Netherlands during a previous study [5] and with a type strain of *M. kansasii* (ATCC 25221) that was kindly provided by Dr. Jakko van Ingen (RadboudUMC, Nijmegen, The Netherlands). The selected strains of *P. aeruginosa*, *S. maltophilia* and *A. fumigatus* were pre-grown in mineral medium with added glucose (0.25 mg/L) and KNO_3_ (6 mg/L) until nutrients were depleted, whereas *M. kansasii* was pre-grown in rich medium.

### 2.2. Experimental Setup

The experimental setup followed the standardized biomass production potential test that was developed to determine the growth potential of pipe materials used in drinking water distribution systems and premises’ plumbing systems (CEN-EN 2014), with some modifications. In total, 12 PVC-P coupons (50 cm^2^ per coupon) were added to 900 mL of drinking water sampled in a bottle from the tap at the KWR laboratory after flushing the tap for 30 min. The drinking water at KWR was produced from groundwater treated with aeration, rapid sand filtration and softening and distributed without a disinfectant residual. This drinking water typically contains 2.4 ng ATP/L and 7.1 × 10^4^ cells/mL. KNO_3_ (60 mg/L) and KH_2_PO_4_ (15 mg/L) were added together with pre-grown *P. aeruginosa*, *S. maltophilia*, *M. kansasii* and *A. fumigatus* to the bottles at day 0 and day 7. This resulted in a mixture of the 4 different species in each bottle, with approximately 10^4^ cells at day 0 and day 7 of each of the added species in each bottle. The inoculum was added at these two time points to enhance the probability that the opportunistic pathogens would colonize the biofilm on the PVC-P coupons. The bottles, with PVC-P coupons and a mixture of the 4 opportunistic pathogens, were subsequently incubated in duplicate at 15.0, 17.5, 20.0, 22.5, 25.0, 27.5 and 30.0 ± 1.0 °C for 112 days. After 14 days of incubation, the drinking water supplemented with KNO_3_ and KH_2_PO_4_ was replaced weekly. On days 0, 7, 14, 28, 56 and 112, 2 PVC-P coupons per bottle (resulting in 4 PVC-P coupons per sampling day per temperature) were taken, and the biofilm from the surface was sampled. The biofilm suspension obtained was used for ATP analysis, cultivation of *P. aeruginosa* and *A. fumigatus*, and the isolation of DNA that was subsequently used for qPCR analyses of *P. aeruginosa*, *S. maltophilia*, *M. kansasii* and *A. fumigatus*.

### 2.3. Microbiological Analyses

The total ATP concentration was determined in all biofilm samples by measuring the amount of light produced in a luciferin–luciferase assay also according to CEN-EN 16421 2014. In short, a nucleotide-releasing buffer (Celsis International B.V., Maastricht, The Netherlands) was added to the sample to release ATP from the cells. The generated light signal was measured as Relative Light Units (RLU) using a luminometer (Celsis Advance II, Celsis International B.V., Maastricht-Airport, The Netherlands). The ATP concentration was calculated from the RLU values using a conversion factor determined from calibration measurements. ATP concentrations were expressed as pg ATP cm^−2^ PVC-P surface.

Cultivable *P. aeruginosa* were determined by plating 0.1 mL of the biofilm suspension sample on *Pseudomonas* agar base supplemented with CN agar, incubated and counted according to ISO standard 16266. Cultivable *A. fumigatus* were determined by plating 0.1 mL of the biofilm suspension sample on malt extract agar, and subsequently, agar plates were incubated for 72 h at 50 °C [22]. After 24, 48 and 72 h, the number of *A. fumigatus* colonies were counted. *P. aeruginosa* and *A. fumigatus* numbers were expressed as colony-forming units (cfu) per square centimeter PVC-P surface.

In total, 30 mL of biofilm suspension was vacuum filtered through polycarbonate track-etch membrane filters with a diameter of 50 mm and a pore size of 0.22 µm (Sartorius; Göttingen, Germany). Filters were transferred to a bead tube of the PowerBiofilm™ DNA isolation kit (MoBio Laboratories Inc., Carlsbad, CA, USA) containing 350 µL of Solution BF1 of the kit. In total, 10 µL of the DNA suspension was used as an internal control to determine the recovery of DNA during extraction and the possible presence of PCR inhibition, as described in standardized protocol ISO 12869:2012. The samples were stored at −20 °C until further processing. The DNA was isolated according to the manufacturer’s protocol, and purified DNA was finally eluted in 200 µL elution buffer from the PowerBiofilm^TM^ kit (Qiagen, Venlo, The Netherlands).

The gene copy numbers of the opportunistic pathogens were determined with previously developed quantitative PCR (qPCR) methods for drinking water samples [4,10,23]. These qPCR methods target the *regA* gene of *P. aeruginosa*, the *chitA* gene of *S. maltophilia*, the intergenic transcribed spacers (ITS) between the 16S and 23S rRNA gene of *M. kansasii* and the 28S rRNA gene of *A. fumigatus.* The reaction mixtures for qPCR analyses contained 25 µL of iQTM SYBR^®^ Green Supermix or iQ™ Supermix (Bio-Rad Laboratories BV, Veenendaal, The Netherlands), 10 µM of each primer and probe, 0.4 mg mL^−1^ bovine serum albumin and 10 µL DNA template in a total volume of 50 µL. Amplification, detection and data analysis were performed in a CFX96TM Real-Time System, C1000TM Thermal Cycler (Bio-Rad Laboratories BV, Veenendaal, The Netherlands). Primer/probe sequences and the amplification programs are shown in Tables S1 and S2. The PCR cycle after which the fluorescence signal of the amplified DNA is detected (threshold cycle [Ct]) was used to quantify the gene copy concentrations. Quantifications were based on a comparison of the sample Ct value with the Ct values of a calibration curve based on known copy numbers of the respective target gene from the different microorganisms. The yield of the added internal control was used to correct the qPCR values for DNA loss during DNA isolation and/or PCR inhibition. The gene copy numbers of each organism were expressed as gene copies (gc) per square centimeter PVC-P surface.

### 2.4. Maximum Biofilm Yield Determination

The maximum biofilm yield of each tested organism was determined by taking the highest average concentration (based on four observations per temperature and per day) measured during the incubation period. The yield is normally expressed as biomass amount per amount of substrate used. In our experiments, we could not determine the substrate concentration because most microbial growth was caused by undefined compounds present in the PVC-P material. However, since the PVC-P coupon area and, consequently, the substrate level was identical between the flasks incubated at different temperatures, we have defined the highest average concentration for each organism in the biofilm (given in cfu per cm^2^ or in gc per cm^2^) as the maximum biofilm yield.

### 2.5. Statistical Analysis

Statistical analysis was performed to test whether the different incubation temperatures resulted in statistically different maximum biofilm yields for the ATP concentration, colony counts of *P. aeruginosa* and *A. fumigatus* and gene copy counts of each tested organism. The colony counts and qPCR data were first log-transformed. Subsequently, an ANOVA test with a Bonferroni post hoc test was conducted on the ATP concentrations and log-transformed colony and gene copy counts to test whether these parameters differed between incubation temperatures. Differences were considered statistically significant when *p* < 0.05.

## 3. Results

### 3.1. Biofilm Growth

**Figure 1:**
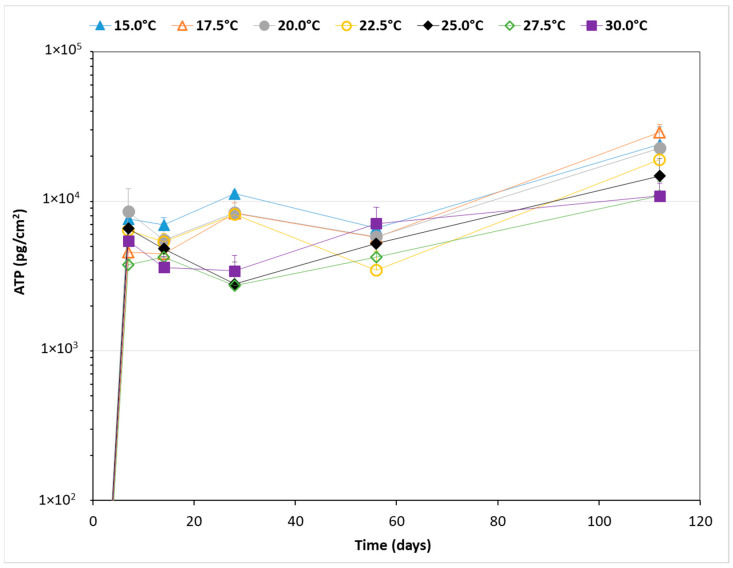
The average ATP concentration ± standard deviation of the biofilm on PVC-P material in contact with drinking water and incubated at seven different temperatures. To keep the graphs readable, only the standard deviation above is shown. On day 0, the ATP concentration was below the detection limit of 10 pg ATP cm^−2^.

The ATP concentration in the biofilm increased by more than a thousand-fold during the first seven days of incubation at all seven incubation temperatures (Figure 1). Thereafter, ATP concentrations remained relatively stable until day 56, followed by a further increase, to ATP concentrations above 1 × 10^4^ pg cm^−2^, from day 56 to 112. The highest ATP concentrations were generally observed at the lowest incubation temperatures (15.0–20.0 °C), and the lowest ATP concentrations were observed at the highest incubation temperatures (25.0–30.0 °C). Moreover, the average ATP concentration ± standard deviation measured in the biofilm at different time points during the incubation period showed a significant and strongly negative correlation with the incubation temperature (*p* < 0.05; R^2^ = 0.92).

### 3.2. Growth of P. aeruginosa, S. maltophilia, M. kansasii and A. fumigatus

**Figure 2:**
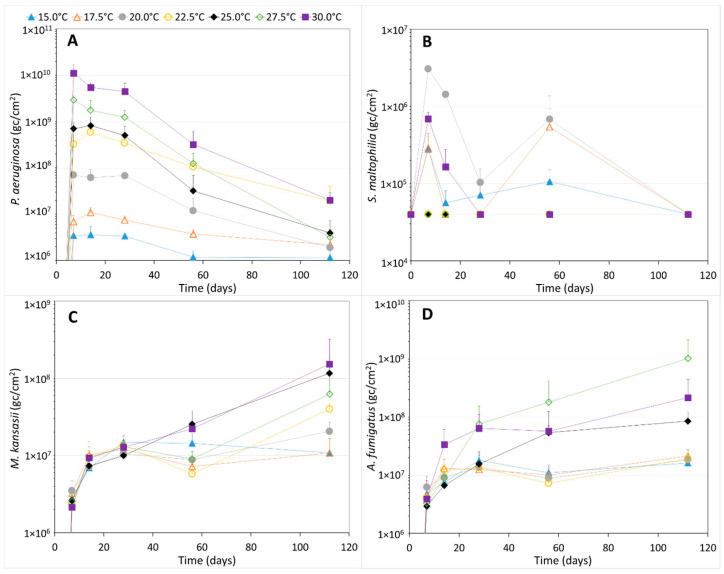
The average gene copy numbers ± standard deviation of *P. aeruginosa* (**A**), *S. maltophilia* (**B**), *M. kansasii* (**C**) and *A. fumigatus* (**D**) in the biofilm on PVC-P material in contact with drinking water and incubated at seven different temperatures. To keep the graphs readable, only the standard deviation above is shown. On day 0, the gene copy numbers of all organisms were below the detection limit of 4 × 10^4^ gc cm^−2^. *S. maltophilia* gene copy numbers were below the detection limit at more time points and are shown as a value of 4 × 10^4^ gc cm^−2^ in graph (**B**).

The gene copy numbers of *P. aeruginosa*, *S. maltophilia*, *M. kansasii* and *A. fumigatus* in the biofilm also increased considerably during the first seven days of incubation at all temperatures (Figure 2). The highest gene copy numbers for *P. aeruginosa* in the biofilm were observed at day 7, 14 or 21, after which the numbers declined substantially until day 112, when gene copy numbers were up to 3 log units lower than at day 7, 14 or 21 (Figure 2A). This suggests that *P. aeruginosa* is among the first colonizers of the biofilm on PVC-P in contact with drinking water and is then replaced by other organisms during biofilm maturation. Moreover, the gene copy numbers of *P. aeruginosa* increased with increasing incubation temperatures. The cultivable *P. aeruginosa* numbers showed the same trends as observed for the *P. aeruginosa* gene copy numbers, except that cultivable numbers were 3.1 to 4.8 log units lower than gene copy numbers (Figure S1A).

The growth of *S. maltophilia* in the biofilm was observed at 15.0, 17.5, 20.0 (only in one of the duplicate bottles) and 30.0 °C. The gene copy numbers at 22.5, 25.0 and 27.5 °C did not increase above the detection limit (4 × 10^4^ gc cm^−2^) (Figure 2B). Since *S. maltophilia* should be capable of growing at 22.5 to 27.5 °C as well (Garrity 2001–2011, Weber et al. 2018), the lack of growth at these 3 temperatures indicates that growth inhibition of *S. maltophilia* occurred. As a result, we cannot rule out that growth at 15.0 to 20.0 °C and 30.0 °C was also partly inhibited. The highest gene copy numbers of *S. maltophilia* were, in general, observed at day 7 or 14 at the 4 temperatures where growth was observed. After 112 days of incubation, the gene copy numbers of *S. maltophilia* in the biofilm were below the detection limit at all incubation temperatures. The highest gene copy numbers were observed at 20.0 and 30.0 °C.

The gene copy numbers of *M. kansasii* in the biofilm on the PVC-P coupons in contact with water and incubated at 15.0 and 17.5 °C increased until day 21, after which numbers stabilized until day 112 (Figure 2C). *M. kansasii* gene copy numbers also increased until day 21 at 20.0, 22.5 and 27.5 °C, and stabilized at these numbers until day 56, after which a second increase from day 56 until 112 was observed. In contrast, gene copy numbers of *M. kansasii* in the biofilm increased during the whole incubation period of 112 days for the bottles incubated at 25.0 and 30.0 °C, indicating that the maximum was not yet reached at the higher incubation temperatures. The highest gene copy numbers were observed at 25.0 and 30.0 °C and the lowest at 15.0 and 17.5 °C. The other incubation temperatures showed numbers in between these highest and lowest values, with numbers increasing with increasing temperature.

The gene copy numbers of *A. fumigatus* in the biofilm incubated at 15.0 to 22.5 °C increased until approximately 14 days, after which the numbers stabilized until day 112 (Figure 2D). In contrast, the gene copy numbers of *A. fumigatus* increased over the whole incubation period when the bottles with PVC-P coupons were incubated at 25.0 to 30.0 °C. The highest numbers were observed at day 112, indicating that the maximum was not yet reached at the three highest temperatures. The highest *A. fumigatus* gene copy numbers in the biofilm were observed at an incubation temperature of 27.5 °C, followed by 30.0 and 25.0 °C. The lowest gene copy numbers were observed at incubation temperatures of 15.0 to 22.5 °C. The cultivable *A. fumigatus* numbers showed the same trends for the PVC-P coupons incubated at 15.0 to 22.5 °C, except that cultivable *A. fumigatus* numbers were 5.3 to 5.7 log units lower than the gene copy numbers (Figure S1B). Cultivable *A. fumigatus* incubated at 25.0 °C were comparable to those incubated at 15.0 to 22.5 °C, which differs from the higher gene copy numbers observed at 25.0 than at 15.0 to 22.5 °C. Consequently, gene copy numbers were up to 5.9 log units higher than cultivable *A. fumigatus* numbers at 25 °C. In contrast to gene copy numbers of *A. fumigatus*, cultivable *A. fumigatus* numbers at 30.0 °C were higher than at 27.5 °C. At these 2 highest incubation temperatures, the gene copy numbers were 5.3 to 6.6 log units higher than the cultivable numbers.

### 3.3. Maximum Biofilm Yields

The results, thus, demonstrated that the water temperature influenced the number of each opportunistic pathogen in drinking water biofilms. To further quantify the effect of water temperature on the growth of *P. aeruginosa*, *M. kansasii* and *A. fumigatus* at each incubation temperature, the maximum biofilm yields were determined for these three pathogens and ATP. The maximum biofilm yield of *S. maltophilia* was not determined because its growth seemed to be inhibited by factors other than the incubation temperature. The maximum ATP biofilm yield of the total microbial biomass in the biofilm varied between 1.1 × 10^4^ and 2.9 × 10^4^ pg ATP cm^−2^, with decreasing concentrations as the temperature increased (Table 1). The maximum ATP biofilm yields at 15.0 and 17.5 °C were significantly higher than those obtained at 27.5 and 30.0 °C (*p* < 0.05).

The maximum biofilm yield of *P. aeruginosa* varied between 3.6 × 10^6^ and 1.1 × 10^10^ gc cm^−2^, which is a much wider range than observed for ATP or the other opportunistic pathogens (Table 1). Furthermore, the maximum biofilm yields of *P. aeruginosa*, determined with qPCR or selective agar plating, showed increasing maximum biofilm yields of *P. aeruginosa* with increasing temperatures (Table 1 and Table S3). The maximum biofilm yield based on gene copies of the *regA* gene of *P. aeruginosa* significantly rose after each increasing incubation temperature step (*p* < 0.05), except between 22.5 and 25.0 °C (Table 1). Similar observations were made for the maximum biofilm yields of cultivable *P. aeruginosa* (Table S3).

The maximum biofilm yield of *M. kansasii* varied between 1.2 × 10^7^ and 1.5 × 10^8^ gc cm^−2^ (Table 1). Relatively low and similar maximum biofilm yields were observed for 15.0 to 20.0 °C, whereas higher incubation temperatures resulted in higher maximum biofilm yields. The maximum biofilm yield observed at 17.5 °C was significantly lower than observed at 25.0 °C (*p* < 0.05), whereas the other maximum biofilm yields for *M. kansasii* were not significantly different between each other (*p* > 0.05).

The maximum biofilm yield of *A. fumigatus* on PVC-P in contact with drinking water was similar when incubated at 15.0 to 22.5 °C (~2.0 × 10^7^ gc cm^−2^; Table 1). The maximum biofilm yield of *A. fumigatus* was higher at the higher incubation temperatures of 25.0 to 30.0 °C (8.6 × 10^7^–1.0 × 10^9^ gc cm^−2^), and the maximum biofilm yield observed at 27.5 °C was significantly higher than those observed at 15.0 to 22.5 °C (*p* < 0.05). The cultivable *A. fumigatus* maximum biofilm yields were also higher when the bottles were incubated at 27.5 and 30.0 °C compared to 15.0 to 22.5 °C, although these differences were not statistically significant (*p* > 0.05) (Table S3).

## 4. Discussion

### 4.1. Influence of Temperature on Opportunistic Pathogens

To determine the influence of drinking water temperature on opportunistic pathogens, we applied the biomass production potential test for pipe materials according to CEN-EN 16241 2014. This test was developed to determine the growth of microorganisms on pipe materials under conditions relevant to drinking water and premises’ plumbing systems [24]. Furthermore, we used PVC-P as pipe material, which is known to have a high growth potential [24,25]. This means that the results obtained are representative for worst-case situations, where drinking water is stagnant and pipe materials support high concentrations of microbial growth.

The results from our study demonstrate that, already at 15.0 °C, cultivable *P. aeruginosa* and *A. fumigatus* numbers increased substantially in the biofilm during the first 7 days of incubation. This increase could have been caused by enhanced attachment of the added microorganisms to the naturally developed biofilm on PVC-P material or by growth. To distinguish between both of these possibilities, we compared the number of cultivable *P. aeruginosa* and *A. fumigatus* that were added at day 0 with those obtained after 7 days of incubation at 15.0 °C. The calculated *P. aeruginosa* numbers on the total PVC-P surface area in the incubation bottles were more than a factor of 10 times higher on day 7 (2.6 × 10^5^ cfu) than the numbers that were added on day 0 to the bottles (1.0 × 10^4^ cfu), demonstrating that *P. aeruginosa* was capable of multiplying in the biofilm at a water temperature of 15 °C. A similar observation was made for *S. maltophilia*.

In contrast, the calculated *A. fumigatus* numbers on the total PVC-P area on day 7 were lower (6.0 × 10^3^ cfu) than the numbers added on day 0 (1.0 × 10^4^ cfu). Thus, it remains uncertain whether *A. fumigatus* is able to multiply at 15.0 °C. Because *A. fumigatus* numbers did not further increase until the temperature reached 25.0 °C (and higher), this implies that *A. fumigatus* was not able to grow at 15.0 to 22.5 °C and that the added *A. fumigatus* cells only attached to and survived in the biofilm during the incubation period at these lower water temperatures. A similar observation was made for *M. kansasii* at 15.0 to 20.0 °C.

In contrast to these opportunistic pathogen numbers, the ATP concentration showed a significant and strong inverse relation with temperature, i.e., the biofilm concentration decreased as temperatures increased. At 30 °C, the active biomass was even 2.7 times lower than at 17.5 °C. An inverse relation between temperature and bacterial biofilm yield was already observed in 1941 by Monod [26] and has been described many times afterwards ([27] and references therein). Both thermodynamic and biochemical factors have been described to explain this inverse relationship ([27] and references therein). Furthermore, an inverse relationship between temperature and the biomass concentration in the biofilm was also previously observed for drinking water systems [28,29]. Consequently, we conclude from these previous studies and our results that the active biomass concentration in the drinking water biofilms decreases when temperatures increase from 15.0 to 30.0 °C.

Prior studies on the influence of temperature on the growth of *P. aeruginosa*, *S. maltophilia* and *A. fumigatus* demonstrated that growth of *P. aeruginosa* occurred between 10 and 42 °C [16], growth of *S. maltophilia* between 10 and 40 °C [20], and growth of *A. fumigatus* between 17 and 56 °C, or 21 to 55 °C, depending on the nutrient medium used for growth [15,19]. These studies were conducted with pure cultures (without competing microorganisms) and under optimal laboratory conditions, in contrast to our study, where the growth of these organisms was determined in biofilms containing an autochthonous microflora. In general, the observed growth of *P. aeruginosa* in these biofilms agrees with the previously observed temperature range for growth with pure cultures, despite the differences in experimental setup. The growth of *S. maltophilia* in the drinking water biofilms observed in our study at different temperatures also agrees to a certain extent with the temperature range for growth observed for pure cultures of *S. maltophilia* [20]. However, in our experiments, *S. maltophilia* was not able to multiply at temperatures between 22.5 and 27.5 °C, temperatures at which pure cultures of *S. maltophilia* were able to grow [20]. A similar observation was made for *A. fumigatus*, which can grow in pure cultures at temperatures lower than 25 °C. This temperature was the lowest temperature where we observed *A. fumigatus* growth in the drinking water biofilm. Possible reasons for which the growth of *S. maltophilia* and *A. fumigatus* did not occur in the biofilm at lower temperatures are that other microorganisms present in the biofilms might possess antagonistic activities against *S. maltophilia* or *A. fumigatus* [30] or outcompete *S. maltophilia* or *A. fumigatus* for nutrients at these temperatures [31]. *A. fumigatus* could be more easily outcompeted at lower temperatures because temperatures between 15 and 25 °C might be suboptimal for the growth of *A. fumigatus*, making it easier for microorganisms that grow optimally at 15 to 25 °C to outcompete *A. fumigatus*.

The observation that some bacterial species outcompeted others at certain temperatures in drinking water systems has been observed for *L. pneumophila* and *L. anisa*. At lower temperatures, *L. anisa* outcompetes *L. pneumophila* in drinking water biofilms, whereas at higher temperatures, the opposite was observed [13].

*P. aeruginosa*, *S. maltophilia*, *M. kansasii* and *A. fumigatus* have been observed in drinking water in many different countries [3,6,32,33,34,35,36,37,38], but drinking water temperatures for these samples were not reported. Previous research in the Netherlands identified the presence of *P. aeruginosa*, *S. maltophilia* and *A. fumigatus* in drinking water predominantly in the summer when temperatures of the drinking water were between 17 and 21 °C [4]. Such temperatures supported the growth of *P. aeruginosa* and *S. maltophilia* in our study. This indicates that the presence of *P. aeruginosa* and *S. maltophilia* in the previously sampled drinking water systems could be cells that actively multiply in the biofilm on the pipe wall or sediment. In addition, the growth of *P. aeruginosa* and *S. maltophilia* in the biofilm that we observed on PVC-P pieces under semi-stagnant conditions in the laboratory could translate to growth under the more dynamic conditions in drinking water distribution systems. This is not surprising, since the experimental setup used in the laboratory was originally developed to predict the amount of growth-promoting compounds in pipe materials under full-scale conditions in drinking water distribution and premises’ plumbing systems [24].

In contrast to the observations for *P. aeruginosa* and *S. maltophilia*, drinking water temperatures of 17 to 21 °C did not result in the growth of *A. fumigatus* in drinking water biofilms under laboratory conditions, although cells or spores of this organism were able to survive at such temperatures. It therefore remains uncertain whether the DNA from *A. fumigatus* that was previously detected in full-scale drinking water systems at moderate temperatures [4] was from actively growing cells, dead cells, resting cells or spores.

Our study focused on drinking water temperatures that occur in drinking water distribution systems in the Netherlands. Most drinking water temperatures there remain below 25 °C [8]. However, 0.1% of the samples exceed the legislative 25 °C standard, but temperatures always remained below 30 °C at those locations. Based on these observations, we chose to use 30 °C as the maximum temperature in our study, although it has been shown that these opportunistic pathogens can also grow at temperatures higher than 30 °C, which are the temperatures that are encountered in premises’ plumbing systems. It is, therefore, recommended to also determine the effect of temperatures between 30 and 60 °C on the growth or decay of these opportunistic pathogens in drinking water biofilms to include the risk of premises’ plumbing systems on the growth of these pathogens.

### 4.2. Impact on Possible Health Risk

Determining the effect of high drinking water temperatures on the growth of opportunistic pathogens in drinking water systems is crucial to elucidate, since increasing drinking water temperatures due to climate change have already been seen and are expected to stay [8,9]. In the Netherlands, drinking water temperatures are routinely monitored at the tap after flushing, and around 42,000 data points are collected each year. The collected data have shown an increasing number of locations where the drinking water temperatures exceed the 25 °C standard of the Drinking Water Decree from 2016 to 2020 [39,40,41,42,43].

The increasing drinking water temperatures could affect the growth of opportunistic pathogens in drinking water systems. In particular, the role of drinking water temperature on the growth of *L. pneumophila* has been studied in the past. Those studies showed that under environmental conditions, *L. pneumophila* seemed to be capable of growing between 25 and 45 °C [44,45,46], although at 25 to 30 °C, *L. pneumophila* was outcompeted by *L. anisa* [13]. *L. anisa* is less pathogenic than *L. pneumophila* and only a health threat to people with very serious underlying disease. The results from these previous studies, thus, have demonstrated that the enhanced growth of *L. pneumophila* in unchlorinated drinking water distribution systems in the Netherlands is only expected when temperatures rise to approximately 30 °C or higher. Such drinking water temperatures are reached in tropical regions [47] but are not likely to occur in temperate climate zones [8]. However, it remains uncertain whether other opportunistic pathogens that have been detected in drinking water systems in the Netherlands (i.e., *P. aeruginosa*, *S. maltophilia*, *M. kansasii* and *A. fumigatus*) [3,4,6,10] could increase when drinking water temperatures increase.

The results from our study showed that the numbers of *P. aeruginosa*, *M. kansasii* and *A. fumigatus* in naturally developed drinking water biofilms increased when incubation temperatures increased from 15.0 to 30.0 °C. We conclude from these results that there is a realistic probability that these three opportunistic pathogens may multiply in drinking water distribution systems if the water temperatures rise due to climate change. Increasing numbers of opportunistic pathogens in the drinking water distribution system can pose an increased public health risk. When high numbers of these organisms reach the plumbing systems of premises, increased growth can be expected, in accordance with the observation that a high inoculum concentration correlates to increased biofilm growth in other ecosystems [48,49]. Exposure of potential patients to a high number (i.e., doses) of these opportunistic pathogens through drinking water can result in higher infection rates (i.e., response) and, consequently, negative public health impact [50,51]. Therefore, we conclude from our results that high drinking water temperatures due to global warming in previously temperate climates can result in an increased public health risk as a result of the opportunistic pathogens investigated in our study.

### 4.3. Impact on Drinking Water Temperature Guidelines and Legislation

The WHO recommends a maximum temperature of 25 °C for drinking water at the tap [11], and a few countries have a regulatory or guideline standard for drinking water temperature [12]. These countries often use the WHO-recommended 25 °C as their temperature standard for drinking water [8]. In the Netherlands, the regulatory standard of 25 °C for drinking water is being discussed because it is increasingly difficult for water utilities to comply with the 25 °C standard during the warm summers experienced over the last years [39,40,41,42,43].

Our results showed that there was a significant and strong correlation between drinking water temperature and the log maximum biofilm yield of *P. aeruginosa* (between 15.0 and 30.0 °C) and *M. kansasii* (between 20.0 and 30.0 °C). These correlations showed that an increase of 1 °C resulted in an increase of 0.22 and 0.052 log units of *P. aeruginosa* and *M. kansasii*, respectively. Furthermore, we observed that *A. fumigatus* was only capable of growing when drinking water temperatures were 25 °C or higher. These results demonstrate that increasing the current drinking water temperature standard above 25 °C can result in higher *P. aeruginosa* and *M. kansasii* numbers and in the growth of *A. fumigatus* in the drinking water system. Consequently, we recommend that countries with a temperate climate use or keep a drinking water temperature of 25 °C as a guideline or legislative standard.

The reduction in drinking water temperature through various methods such as deep installation of distribution pipes underground, applying a minimum distance between district heating pipes or electricity cables and drinking water pipes underground and ground shading by vegetation or shade structures above distribution pipes underground are possible control measures [8], although additional research is required to investigate the efficacy of these approaches.

## 5. Conclusions

- The active biomass concentration in the drinking water biofilms decreases when temperatures increase from 15.0 to 30.0 °C.
- There is a realistic probability that the numbers of *P. aeruginosa*, *M. kansasii* and *A. fumigatus* will increase in drinking water distribution systems in temperate climates as drinking water temperatures rise due to climate change, posing an increased public health risk.
- Increasing the current drinking water temperature standard above the WHO guideline standard of 25 °C can result in higher *P. aeruginosa* and *M. kansasii* numbers and in the higher growth of *A. fumigatus* in drinking water systems than numbers occurring at temperatures below 25 °C. As a result, changing this guideline is not recommended.

## Figures and Tables

**Table 1:** The maximum growth yield (average ± standard deviation of four replicates) based on the ATP concentration and the gene copies of *P. aeruginosa*, *M. kansasii* and *A. fumigatus* in the biofilm on PVC-P in contact with drinking water at different temperatures. Different letters in each column denote significant differences in each parameter between different incubation temperatures (ANOVA, Bonferroni post hoc, *p* < 0.05).

**Temperature** **(°C)**	**ATP** **(pg cm^−2^)**	***P. aeruginosa*** **(gc cm^−2^)**	***M. kansasii*** **(gc cm^−2^)**	***A. fumigatus*** **(gc cm^−2^)**
15.0	2.4 ± 0.7 × 10^4 a^	3.6 ± 1.9 × 10^6 a^	1.5 ± 0.3 × 10^7^	1.8 ± 0.4 × 10^7 a^
17.5	2.9 ± 0.4 × 10^4 a^	1.1 ± 0.3 × 10^7 b^	1.2 ± 0.7 × 10^7 a^	2.1 ± 0.5 × 10^7 a^
20.0	2.3 ± 0.6 × 10^4^	7.1 ± 1.4 × 10^7 c^	2.1 ± 0.6 × 10^7^	1.8 ± 1.0 × 10^7 a^
22.5	1.9 ± 0.5 × 10^4^	5.9 ± 1.1 × 10^8 d^	4.0 ± 0.7 × 10^7^	1.9 ± 0.4 × 10^7 a^
25.0	1.5 ± 0.5 × 10^4^	8.2 ± 4.3 × 10^8 d^	1.2 ± 0.7 × 10^8 b^	8.6 ± 3.7 × 10^7^
27.5	1.1 ± 0.3 × 10^4 b^	2.9 ± 0.7 × 10^9 e^	6.3 ± 5.5 × 10^7^	1.0 ± 1.1 × 10^9 b^
30.0	1.1 ± 0.2 × 10^4 b^	1.1 ± 0.6 × 10^10 f^	1.5 ± 1.7 × 10^8^	2.2 ± 2.3 × 10^8^

## Data Availability

All data and standard operation procedures are provided in the manuscript or the Supplementary Materials.

## Supplementary Materials

The following supporting information can be downloaded at https://www.mdpi.com/article/10.3390/microorganisms11061574/s1, Figure S1: The average cultivable *P. aeruginosa* (A) and *A. fumigatus* (B) numbers ± standard deviation in the biofilm on PVC-P material in contact with drinking water and incubated at seven different temperatures; Table S1: PCR primers used in this study; Table S2: PCR amplification programs for the different species; Table S3: The maximum growth yield (average ± standard deviation of four replicates) for the colony-forming units of *P. aeruginosa* and *A. fumigatus* in the biofilm on PVC-P in contact with drinking water at different temperatures. Different letters in each column denote significant differences in max yield between temperatures (ANOVA, Bonferroni post hoc, *p* < 0.05). References [10,23,52,53] are cited in the supplementary materials.
