# Supplementary material for: Influence of Temperature on Growth of Four Different Opportunistic Pathogens in Drinking Water Biofilms
Source: Microorganisms. 2023 Jun 14;11(6):1574. doi: 10.3390/microorganisms11061574 (PMC10303289; doi:10.3390/microorganisms11061574)
Supplement: Supplementary file 1 [file microorganisms-11-01574-s001.zip › microorganisms-2407785-supplementary.pdf]

Supplementary Materials

Influence of Temperature on Growth of Four Different Opportunistic Pathogens in Drinking Water Biofilms

Paul W. J. J. van der Wielen [1,2,*], Marco Dignum [3], Agata Donocik [4] and Emmanuelle I. Prest [5]

[1] KWR Water Research Institute, Nieuwegein, The Netherlands; paul.van.der.wielen@kwrwater.nl
[2] Laboratory of Microbiology, Wageningen University & Research, Wageningen, The Netherlands
[3] Waternet, Amsterdam, The Netherlands; marco.dignum@waternet.nl
[4] Brabant Water, Den Bosch, The Netherlands; agata.donocik@brabantwater.nl
[5] PWN Technologies, Andijk, The Netherlands; emmanuelle.prest@pwnt.com
* Correspondence: paul.van.der.wielen@kwrwater.nl

Table S1 PCR primers used in the study

Microorganism	Primers	Reference
P. aeruginosa	Forward: 5′-ATCGAGTACCTGAACCGGC-3′	(1)
	Reverse: 5′-TGGTGCAGTTCCTCATTGTC-3′	
	Probe: 5′-CCAGATGCTTTGCCTCAAC-3′	
S. maltophilia	Forward: 5′-TACCACCCGTACCTGGACTT-3′	(2)
	Reverse: 5′-ATCGCATCGTTGCTGTTGTA-3′	
M. kansasii	Forward: 5′-CGAAAAGCATCCCAACAAGTGG-3′	
	Reverse: 5′-GTGGGACAACTCTCGAACAG -3′	(3)
	Probe: 5′-TCTGTAGTGGACGAAAGCCGGG-3′	
A. fumigatus	Forward: 5′-CTCGGAATGTATCACCTCTCGG-3′	(4)
	Reverse: 5′-TCCTCGCTCCAGGCAGG-3′	
	Probe: 5′-TGTCTTATAGCCGAGGGTGCAATGCG-3′	

Table S2 PCR amplification programs for the different species

Microorganism	PCR program
P. aeruginosa	2 min 95°C; 43 cycles: 20 sec 95°C, 1 min 60°C
S. maltophilia	5 min 95°C; 43 cycles: 30 sec 95°C, 30 sec 58°C, 1 min 72°C; 10 min 72°C
M. kansasii	5 min 95°C; 43 cycles: 20 sec 95°C, 48 sec 60°C
A. fumigatus	10 min 95°C; 43 cycles: 15 sec 95°C, 1 min 60°C

Table S3 The maximum growth yield (average ± standard deviation of four replicates) for the colony forming units of *P. aeruginosa* and *A. fumigatus* in the biofilm on PVC-P in contact with drinking water at different temperatures. Different letters in each column denotes significant differences in max yield between temperatures (ANOVA, Bonferroni post-hoc, $p<0.05$).

Temperature	*P. aeruginosa* (cfu cm^{-2})	*A. fumigatus* (cfu cm^{-2})
15.0	$7.6 \pm 1.7 \times 10^{2}$ [a]	60 ± 16
17.5	$6.7 \pm 6.2 \times 10^{3}$ [b]	80 ± 19
20.0	$1.4 \pm 0.5 \times 10^{4}$ [b]	56 ± 14
22.5	$1.1 \pm 0.2 \times 10^{5}$ [c]	66 ± 18
25.0	$3.2 \pm 2.3 \times 10^{5}$ [c]	99 ± 29
27.5	$4.7 \pm 1.6 \times 10^{5}$ [c, d]	260 ± 240
30.0	$2.1 \pm 0.7 \times 10^{6}$ [d, e]	540 ± 557

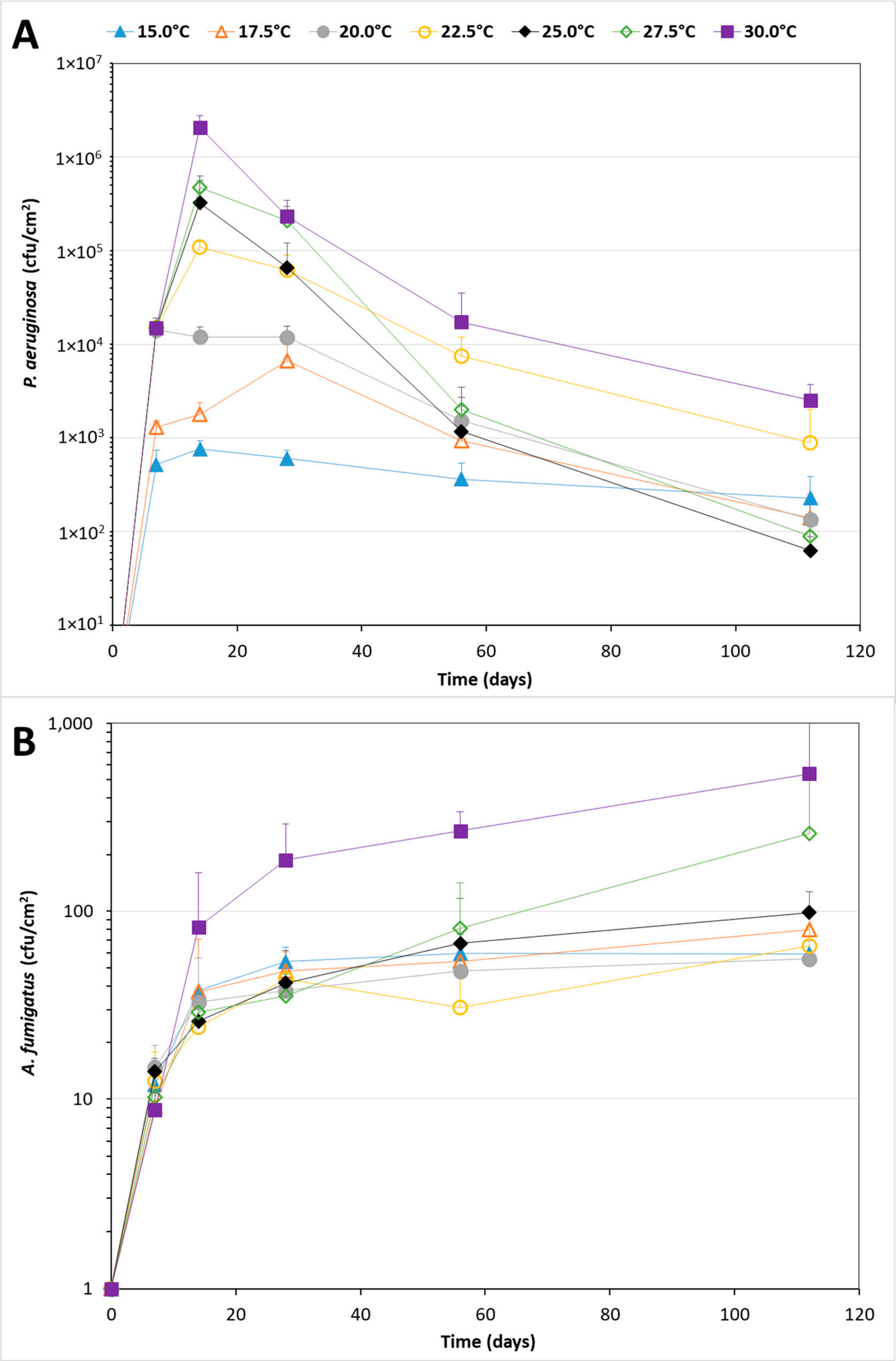

Figure S1. The average cultivable *P. aeruginosa* (A) and *A. fumigatus* (B) numbers ± standard deviation in the biofilm on PVC-P material in contact with drinking water and incubated at seven different temperatures. To keep the graphs readable only the standard deviation above is shown.

REFERENCES

1. van der Wielen PWJJ, Italiaander R, Wullings BA, Heijnen L, van der Kooij D. 2014. Opportunistic pathogens in drinking water in the Netherlands, p 177-205. *In* van der Kooij D, van der Wielen PWJJ (ed), Microbial growth in drinking-water supplies. Problems, causes, control and research needs. IWA Publishing, London, UK.

2. da Silva Filho LV, Tateno AF, de Velloso FL, Levi JE, Fernandes S, Bento CN, Rodrigues JC, Ramos SR. 2004. Identification of *Pseudomonas aeruginosa*, *Burkholderia cepacia* complex, and *Stenotrophomonas maltophilia* in respiratory samples from cystic fibrosis patients using multiplex PCR. Pediatr. Pulmonol. 37:537-547.

3. van der Wielen PWJJ, Uytewaal-Aarts, M. 2013. Non-tuberculous mycobacteria in drinking water (in Dutch). KWR Water Research Institute, Nieuwegein, the Netherlands.

4. Challier S, Boyer S, Abachin E, Berche P. 2004. Development of a serum-based Taqman real-time PCR assay for diagnosis of invasive aspergillosis. J Clin Microbiol 42:844-846.
